# Cardiac autonomic function and cognitive performance in patients with atrial fibrillation

**DOI:** 10.1007/s00392-021-01900-4

**Published:** 2021-06-22

**Authors:** Peter Hämmerle, Stefanie Aeschbacher, Anne Springer, Ceylan Eken, Michael Coslovsky, Gilles Dutilh, Giorgio Moschovitis, Nicolas Rodondi, Patricia Chocano, David Conen, Stefan Osswald, Michael Kühne, Christine S. Zuern

**Affiliations:** 1 Department of Cardiology, Cardiovascular Research Institute Basel, University Hospital Basel, Basel, Switzerland; 2 Department Clinical Research, University of Basel, Basel, Switzerland; 3 Department of Cardiology, Hospedale Regionale Di Lugano, Lugano, Switzerland; 4 Institute of Primary Health Care (BIHAM), University of Bern, Bern, Switzerland; 5 Department of General Internal Medicine, Inselspital, Bern University Hospital, Bern, Switzerland; 6 Population Health Research Institute, McMaster University and Hamilton Health Sciences, Hamilton, Canada; 7 Department of Cardiology, University Hospital Basel, Basel, Switzerland

**Keywords:** Atrial fibrillation, Cardiac autonomic function, Heart rate variability, Neurocognitive function, Montreal Cognitive Assessment

## Abstract

**Background:**

Atrial fibrillation (AF) is associated with loss of cognition and dementia. Cardiac autonomic dysfunction has been linked to cognitive decline. We aimed to investigate if reduced cardiac autonomic function (CAF) is associated with cognitive impairment in AF patients.

**Methods:**

Patients with paroxysmal, persistent and permanent AF were enrolled from a multicenter cohort study if they had AF (“AF group”) or sinus rhythm (“SR group”) on a baseline 5 min ECG recording. Parameters quantifying CAF (heart rate variability triangular index (HRVI), mean heart rate (MHR), RMSSD, SDNN, total power and power in the VLF, LF, HF ranges) were calculated. We used the Montreal Cognitive Assessment (MoCA) to assess global cognitive function.

**Results:**

1685 AF patients with a mean age of 73 ± 8 years, 29% females, were included. MoCA score was 24.5 ± 3.2 in the AF group (*N *= 710 patients) and 25.4 ± 3.2 in the SR group (*N *= 975 patients). After adjusting for multiple confounders, lower HRVI was associated with lower MoCA scores, both in the SR group [β = 0.049; 95% confidence interval (CI) 0.016–0.081; *p* = 0.003] and in the AF group (β = 0.068; 95% CI 0.020–0.116; *p *= 0.006). In the AF group, higher MHR was associated with a poorer performance in the MoCA (β =  − 0.008; 95% CI − 0.014 to − 0.002; *p *= 0.014). We found no convincing evidence of association for other CAF parameters with cognition.

**Conclusion:**

Our data suggest that impaired CAF is associated with worse cognitive performance in patients with AF. Among standard HRV parameters, HRVI might be the most promising ECG index.

**Trial registration:**

ClinicalTrials.gov Identifier: NCT02105844.

**Graphic abstract:**

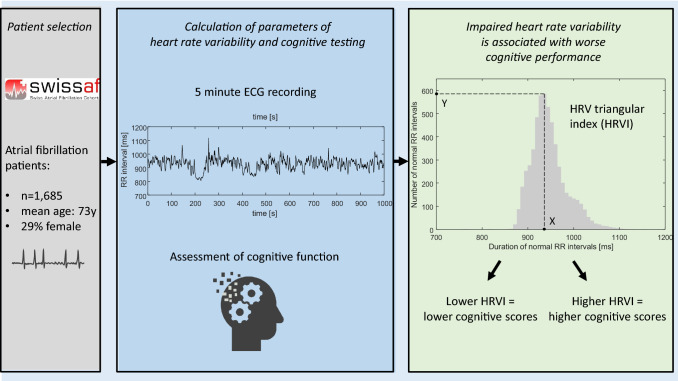

**Supplementary Information:**

The online version contains supplementary material available at 10.1007/s00392-021-01900-4.

## Introduction

Cognitive impairment is a global health burden and imposes a major challenge to affected individuals, families and healthcare systems. Among numerous risk factors, atrial fibrillation (AF) is strongly associated with cognitive decline [1–5]. Structural brain lesions, including (clinically unrecognized) brain infarcts, white matter lesions and microbleeds have been reported to contribute to cognitive dysfunction in AF patients [6, 7].

Cardiac autonomic function (CAF) has been related to cognitive performance in sinus rhythm (SR) patients [8–13]. CAF can be quantified by means of heart rate variability (HRV) derived from Holter ECG but also from standard ECG recordings [14]. A cross-sectional analysis of a general Irish population revealed that lower quintiles of the standard deviation of the normal-to-normal intervals (SDNN), low frequency (LF), and LF:high frequency (HF) ratio were associated with lower Montreal Cognitive Assessment (MoCA) scores [8]. Another study showed that impaired HRV was related to worse performance in the Modified Mini-Mental State Examination in elderly Mexican Americans [10].

However, previous studies investigating the link between CAF and cognition have excluded AF patients due to the challenges of quantifying CAF in AF. Therefore, the association between HRV and cognitive performance in patients with AF is still unexplored. As AF is related to an increased risk of cognitive impairment [1–4], quickly and automatically available parameters that may help to screen for cognitive dysfunction in AF patients are of great clinical interest.

The purpose of this investigation was to examine whether HRV is associated with global cognitive performance in patients with AF, assessed by the MoCA [15].

## Methods

### Study population

The present analysis includes patients from the Swiss Atrial Fibrillation Cohort (Swiss-AF), an ongoing prospective, observational, multicenter cohort study (ClinicalTrials.gov Identifier: NCT02105844). In Swiss-AF, 2415 patients with documented AF were enrolled across 14 centers in Switzerland between 2014 and 2017. The detailed methodology has been described elsewhere [16]. Main inclusion criteria were previously documented AF and age ≥ 65 years. Exclusion criteria were short secondary, reversible episodes of AF (e.g. after cardiac surgery), acute illness within the last 4 weeks or if patients were unable to sign the informed consent. The study protocol was approved by the local ethic committees, and written informed consent was obtained from each participant.

**Fig. 1:**
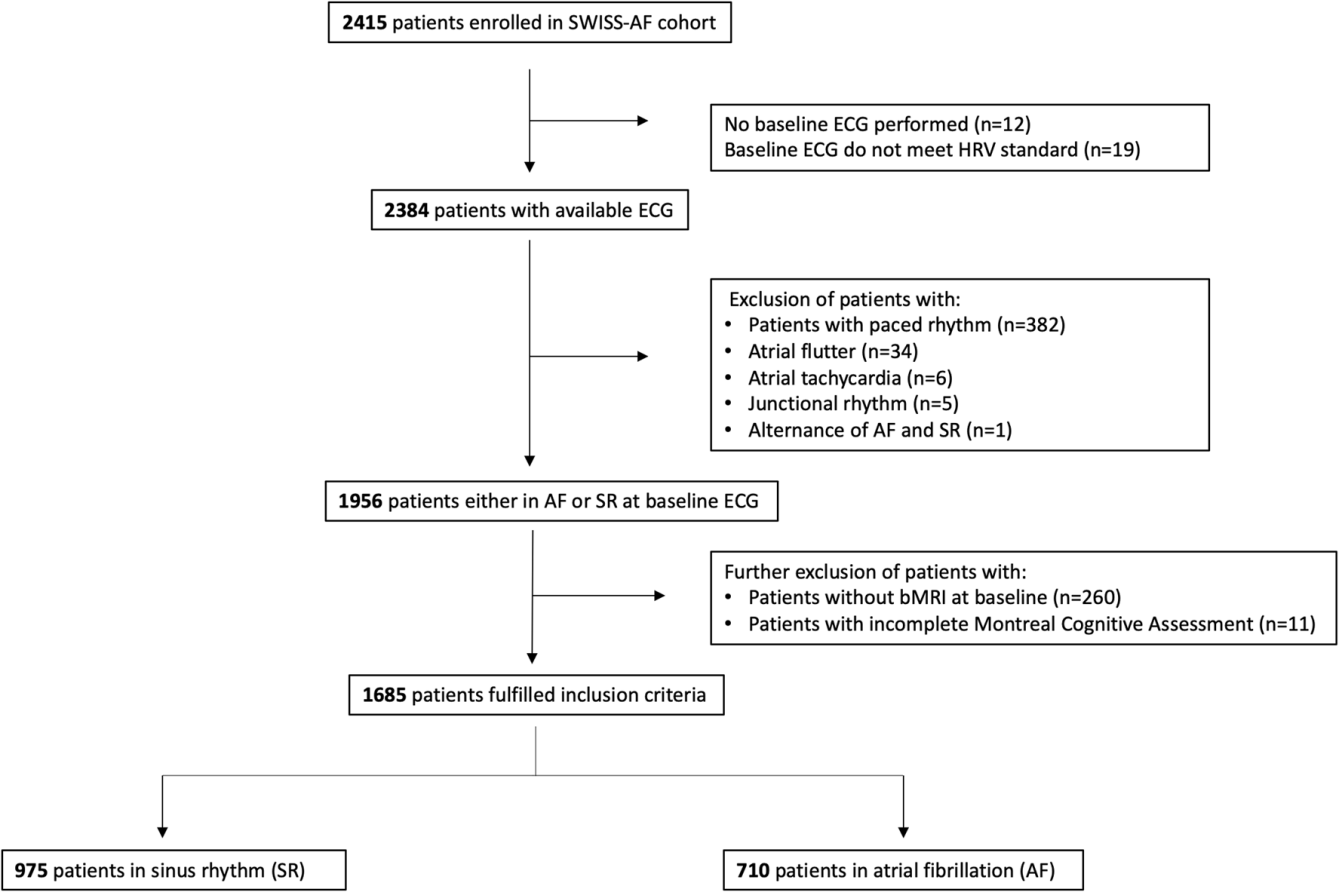
Flow chart of patient selection

For this analysis, we had to exclude a total of 730 Swiss-AF patients: 12 due to missing baseline ECGs, 19 due to low quality ECGs, 428 due to rhythms other than SR or AF (e.g. paced rhythms, atrial flutter), 260 without brain magnetic resonance imaging (bMRI), and 11 due to incomplete MoCA. Thus, 1685 Swiss-AF patients remained for this analysis, of whom 975 had SR (“SR group”) and 710 had AF (“AF group”) on the baseline ECG recording (Fig. 1).

### Study variables

Information on individual patient characteristics, medical history and current medication was collected using standardized case report forms. Blood pressure was measured three times in supine position and the mean was calculated for this analysis. AF type was categorized as paroxysmal, persistent and permanent according to the guidelines of the European Society of Cardiology [17]. Education level was indicated using standardized questionnaires, differentiating between basic educational level (less than 6 years of school), middle educational level (7–11 years of school) and advanced educational achievement (12 or more years of school). Brain MRI was performed at each participating site on a 1.5 or 3 Tesla scanner, to detect structural brain lesions, including large non-cortical or cortical infarcts. These infarcts were defined as either hyper-intense lesions on FLAIR > 20 mm in diameter on axial sections not involving the cortex or of any infarcts involving the cortex. All images were analyzed in a core lab by trained MRI technicians and validated by board-certified radiologists. Brain lesions were evaluated using the AMIRA software. The detailed methodology of the bMRI protocol has been described previously [7].

### Assessment of cardiac autonomic function

16-lead resting ECGs (the standard 12 leads plus 2 additional precordial leads on the right side and 2 on the back) of a duration of 5 min were recorded at baseline in each participant (CS-200 Excellence and CS-200 Touch, Schiller AG, Baar, Switzerland). We saved all ECGs digitally with a sampling frequency of 1 kHz (signal bandwidth 0.04–387 Hz) and a resolution of 1 µV/bit on a central server. The following parameters of HRV were calculated according to previously published algorithms [18]: heart rate variability triangular index (HRVI), mean heart rate (MHR), root mean square of successive differences (RMSSD) and SDNN. We performed spectral analysis to calculate frequency domain measures of HRV: 5-min total power, power in the very low frequency range (VLF, ≤ 0.04 Hz), in the LF range (0.04–0.15 Hz) and in the HF range (0.15–0.4 Hz).

### Cognitive testing

All study personnel underwent standardized training on how to conduct cognitive assessments in the study participants. To ensure continuous data quality, experienced staff study personnel from the University of Basel centrally trained each new investigator at the respective study center. We performed cognitive testing using the MoCA score, which is a validated global cognitive assessment for screening of mild cognitive impairment [15] on the same day as the ECG recording. The total test score ranges from 0 to 30, reflecting cognitive capabilities in various domains, such as short-term memory, visuospatial capacity (e.g. clock-drawing), language (e.g. animal naming task) and orientation. Higher scores indicate better cognitive performance. Moreover, different areas of executive functions are measured, including attention, mental flexibility and working memory. Participants received one additional point if they had a MoCA score < 30 and 12 years or less of formal school education.

### Statistical analysis

Baseline characteristics were stratified by the presence of SR or AF on baseline ECG recording. Numbers are presented as counts (percentage) for categorical variables, means (± standard deviation) for normally and medians (interquartile range) for not normally distributed continuous variables. Groups are compared using a Chi-square test for categorical variables and Student’s *t* test or Mann–Whitney *U* test for continuous variables.

To investigate the association between CAF and MoCA, we performed multivariable adjusted linear regression analyses; we report the β-coefficients and the corresponding 95% confidence intervals (CI). The multivariable models were adjusted for a pre-defined set of potential confounders (age, sex, body mass index (BMI), smoking status (current/past vs. never), alcohol consumption [19], presence of large non-cortical or cortical infarcts, history of hypertension, history of diabetes, education (basic, middle, advanced), history of oral anticoagulation therapy, intake of beta blockers and/or class Ic/III antiarrhythmics). As a sensitivity analysis for slight ceiling effects associated with the MoCA, we repeated all analyses via a Tobit regression (supplemental material). In addition, we built an age-adjusted linear regression model to assess the effect of baseline rhythm (SR vs. AF) on cognition (MoCA). Frequency domain measures of HRV were log-transformed. All analyses were stratified by baseline rhythm. Due to the exploratory nature of this analysis, we did not correct p values and CIs for multiple testing. Statistical analyses were done using SPSS (IBM Corp., Version 22).

## Results

Baseline characteristics for the SR group and AF group (according to rhythm on baseline ECG) are presented in Table 1. Mean age of the study population was 73 ± 8 years and 29% were female. Patients in the SR group were younger (71 ± 8 years vs 75 ± 8 years), had a lower prevalence of co-morbidities, such as hypertension (65% vs 75%), diabetes (13% vs 19%), prior myocardial infarction (11% vs 17%) and heart failure (15% vs 32%). The median CHA_2_DS_2_-Vasc-Score was 3.2 in the SR group and 3.8 in the AF group. SR group-patients were more often treated with non-Vitamin K oral anticoagulants (62% vs 42%) and less often with Vitamin K antagonists (25% vs 53%), received more often class Ic/III anti-arrhythmics (30% vs 24%) and less often beta blockers (65% vs 71%). More patients in the SR group had undergone electrical cardioversion (37% vs 33%) and pulmonary vein isolation (34% vs 6%) compared to patients in the AF group. Large non-cortical or cortical infarcts were detected in 16.7% of patients in the SR group, with a median volume of 954 mm^3^. In the AF group, large non-cortical or cortical infarcts were found in 29.3% (median volume 2679 mm^3^).

**Table 1:** Baseline characteristics of the patients stratified by baseline rhythm

Characteristic	Sinus rhythm group (*N *= 975)	Atrial fibrillation group (*N *= 710)	*p* value*
Age, years	71 ± 8	75 ± 8	< 0.001
Female sex, *N* (%)	295 (30)	188 (26)	0.003
Body mass index, kg/m^2^	27.2 ± 4.9	28.2 ± 4.9	< 0.001
Blood pressure, mm Hg	137 ± 18/78 ± 11	133 ± 19/ 80 ± 13	0.001/0.002
History of hypertension, *N* (%)	632 (65)	533 (75)	< 0.001
History of diabetes mellitus, *N* (%)	123 (13)	135 (19)	< 0.001
Active and former smokers, *N* (%)	545 (56)	400 (56)	0.839
History of electrocardioversion, *N* (%)	364 (37)	234 (33)	0.064
History of pulmonary vein isolation, *N* (%)	335 (34)	51 (6)	< 0.001
History of myocardial infarction, *N* (%)	107 (11)	118 (17)	0.001
History of heart failure, *N* (%)	142 (15)	226 (32)	< 0.001
History of stroke/TIA, N (%)	164 (17)	171 (24)	< 0.001
CHA_2_DS_2_-VASc score, points	3.2 ± 4.3	3.8 ± 1.7	< 0.001
Paroxysmal atrial fibrillation, *N* (%)	654(67)	110 (15)	< 0.001
Persistent atrial fibrillation, *N* (%)	321 (33)	210 (30)	0.144
Permanent atrial fibrillation, *N* (%)	–	390 (55)	–
Antiarrhythmic therapy (class Ic and III), *N* (%)	293 (30)	173 (24)	0.010
Beta-blockers, *N* (%)	631 (65)	501 (71)	0.012
Non vitamin K oral anticoagulants, *N* (%)	602 (62)	295 (42)	< 0.001
Vitamin K antagonists, *N* (%)	241 (25)	376 (53)	< 0.001

All patients had a diagnosis of atrial fibrillation. Data are means ± SD or counts (percentages)

*p value compares sinus rhythm and atrial fibrillation groups and were obtained from Student’s *t* tests for continuous variables and chi-square tests for categorical variables

**Fig. 2:**
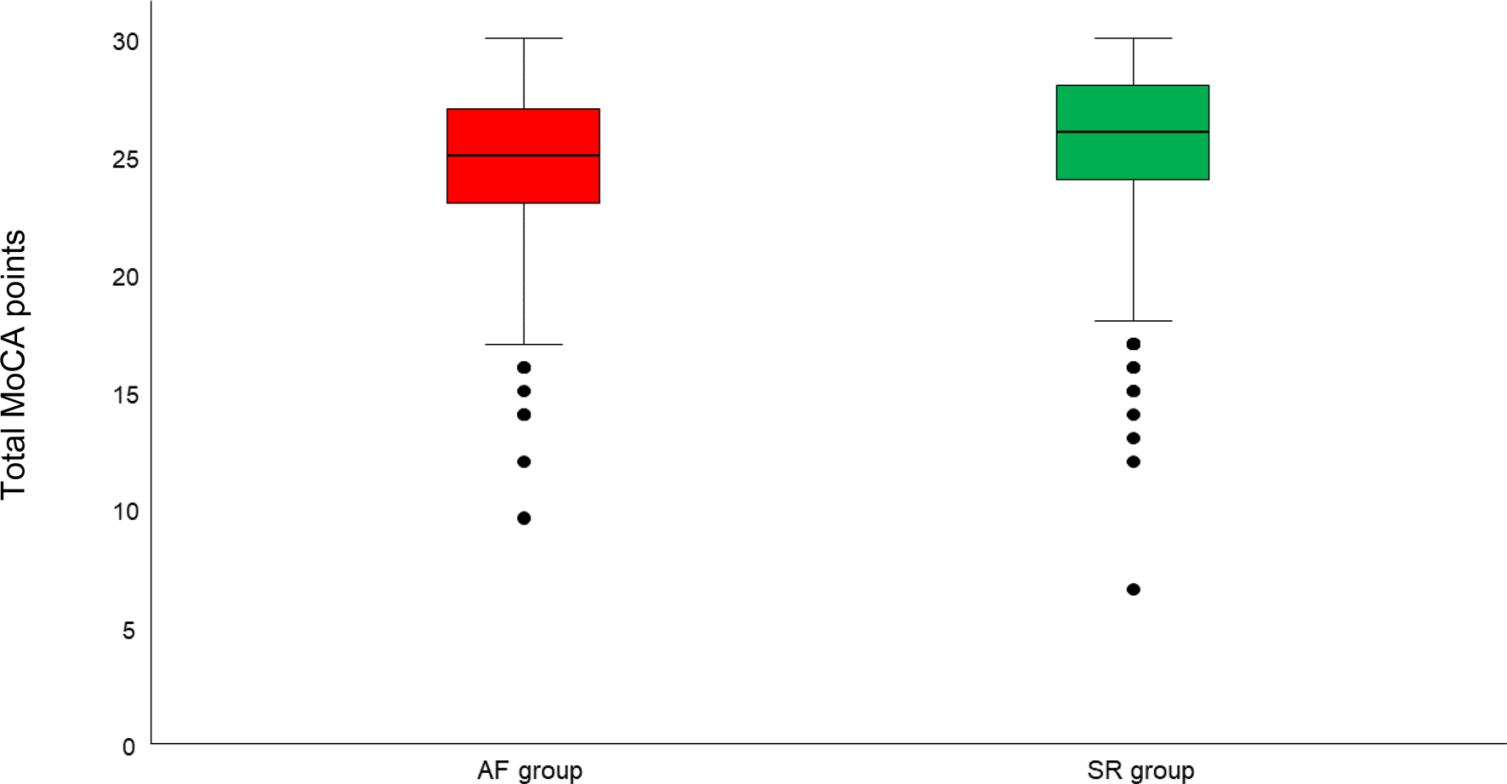
Cognitive performance assessed by the MoCA

Table 2 shows the results of time and frequency domain measures of HRV in the SR group and AF group. All HRV measures were higher in the AF group (*p *< 0.05 for all). With regard to cognitive performance, patients in the SR group achieved higher MoCA scores (median 26 (IQR 24–28) vs. median 25 (23–27), *p *< 0.001, Fig. 2) compared to the AF group, which persisted after adjustment for age (β-coefficient = 0.438; 95% CI 0.131–0.746; *p *= 0.005).

**Table 2:** Parameters of heart rate variability in patients stratified by baseline rhythm

HRV parameter	Sinus rhythm group (*N *= 975)	Atrial fibrillation group (*N *= 710)	*p* value*
HRVI	14.4 (11.9–18.05)	15.3 (12.7–18.7)	< 0.001*
MHR	86.4 (45.0–145.1)	101 (77.7–134)	< 0.001*
SDNN	35.7 (20.8–46.9)	53.9 (40.3–74.7)	< 0.001*
RMSSD	85.4(63.4–143.3)	127 (86.8–152)	< 0.001*
5 min total power^b^	3.4 ± 0.8	3.7 ± 0.3	< 0.001^a^
HF^b^	2.8 ± 0.7	3.1 ± 0.3	< 0.001^a^
LF^b^	3.2 ± 0.8	3.5 ± 0.3	< 0.001^a^
VLF^b^	3.1 ± 0.9	3.3 ± 0.6	< 0.001^a^

Data are medians and interquartile ranges or means and standard deviations

*HF* high frequency, *HRVI* heart rate variability triangular index, *LF* low frequency, *MHR* mean heart rate, *RMSSD* square root of the mean squared differences of successive normal-to-normal intervals, *SDNN* standard deviation of the normal-to-normal intervals, *VLF* very low frequency

*p value compares sinus rhythm and atrial fibrillation groups and was obtained from Mann–Whitney-Test* or from Student’s *t* test^a^

^b^Frequency domain measures of heart rate variability were log-transformed

Results of the associations between CAF and MoCA are presented in Tables 3 and 4. After adjustment for age, sex, BMI, smoking status, alcohol consumption, presence of large non-cortical or cortical infarcts, history of hypertension, diabetes, education, oral anticoagulation, beta blockers and/or class Ic/III antiarrhythmics, lower HRVI was associated with lower MoCA scores in the SR group (β-coefficient = 0.049; 95% CI 0.016–0.081; *p *= 0.003). Furthermore, in the AF group, lower HRVI was related to lower MoCA scores (β-coefficient = 0.068; 95% CI 0.020–0.116; *p *= 0.006). When additionally adjusted for pre-defined co-variables (AF duration, intake of non-dihydropyridine calcium channel blockers and digoxin), lower HRVI was still associated with lower MoCA scores in the SR and AF group (Supplemental Table 1). The results did not change substantially when using a Tobit regression (Supplemental Table 2).

**Table 3:** Associations between time domain measures of HRV and the study endpoint (MoCA)

HRV parameter	Sinus rhythm group			Atrial fibrillation group		
	β (95% CI)	*p* value*	*R*^2^	β (95% CI)	*p* value*	*R*^2^
HRVI	0.049 (0.016; 0.081)	0.003	0.175	0.068 (0.020; 0.116)	0.006	0.184
MHR	− 0.003 (− 0.007; 0.002)	0.254	0.169	− 0.008 (− 0.014; − 0.002)	0.014	0.183
RMSSD	− 0.008 (− 0.018; 0.001)	0.089	0.170	− 0.006 (− 0.016; 0.003)	0.174	0.178
SDNN	0.001 (− 0.002; 0.003)	0.566	0.168	− 0.001 (− 0.006; 0.004)	0.681	0.176

Data are beta-coefficients (β) [95% confidence intervals (CI)]

*HRV* heart rate variability, *HRVI* heart rate variability triangular index, *MoCA* Montreal Cognitive Assessment, *MHR* mean heart rate, *RMSSD* square root of the mean squared differences of successive normal-to-normal intervals, *SDNN* standard deviation of the normal-to-normal intervals

*p values were based on linear regression models. Multivariable model was adjusted for age, sex, body mass index, smoking status (current/past vs. never), alcohol consumption, presence of large non-cortical or cortical infarcts, history of hypertension, history of diabetes, education (basic, middle, advanced), history of oral anticoagulation therapy, intake of beta blockers and/or class Ic/III antiarrhythmics

**Table 4:** Associations between frequency domain measures of HRV and the study endpoint (MoCA)

HRV parameter	Sinus rhythm group			Atrial fibrillation group		
	β (95% CI)	*p* value*	*R*^2^	β (95% CI)	*p* value*	*R*^2^
5 min total power^a^	0.100 (− 0.132; 0.322)	0.396	0.166	− 0.113 (− 0.847; 0.622)	0.764	0.176
HF^a^	0.047 (− 0.192; 0.287)	0.699	0.166	− 0.007 (− 0.648; 0.634)	0.982	0.175
LF^a^	0.009 (− 0.210; 0.228)	0.937	0.166	− 0.187 (− 0.825; 0.451)	0.564	0.176
VLF^a^	0.036 (− 0.182; 0.254)	0.744	0.169	− 0.288 (− 0.642; 0.066)	0.110	0.182

Data are beta-coefficients (β) [95% confidence intervals (CI)]

*HF* high frequency, *HRV* heart rate variability, *LF* low frequency, *MoCA* Montreal Cognitive Assessment, *VLF* very low frequency

*p values were based on linear regression models. Multivariable model was adjusted for age, sex, body mass index, smoking status (current/past vs. never), alcohol consumption, presence of large non-cortical or cortical infarcts, history of hypertension, history of diabetes, education (basic, middle, advanced), history of oral anticoagulation therapy, intake of betablockers and/or class Ic/III antiarrhythmics

^a^Frequency domain measures of heart rate variability were log-transformed

**Fig. 3:**
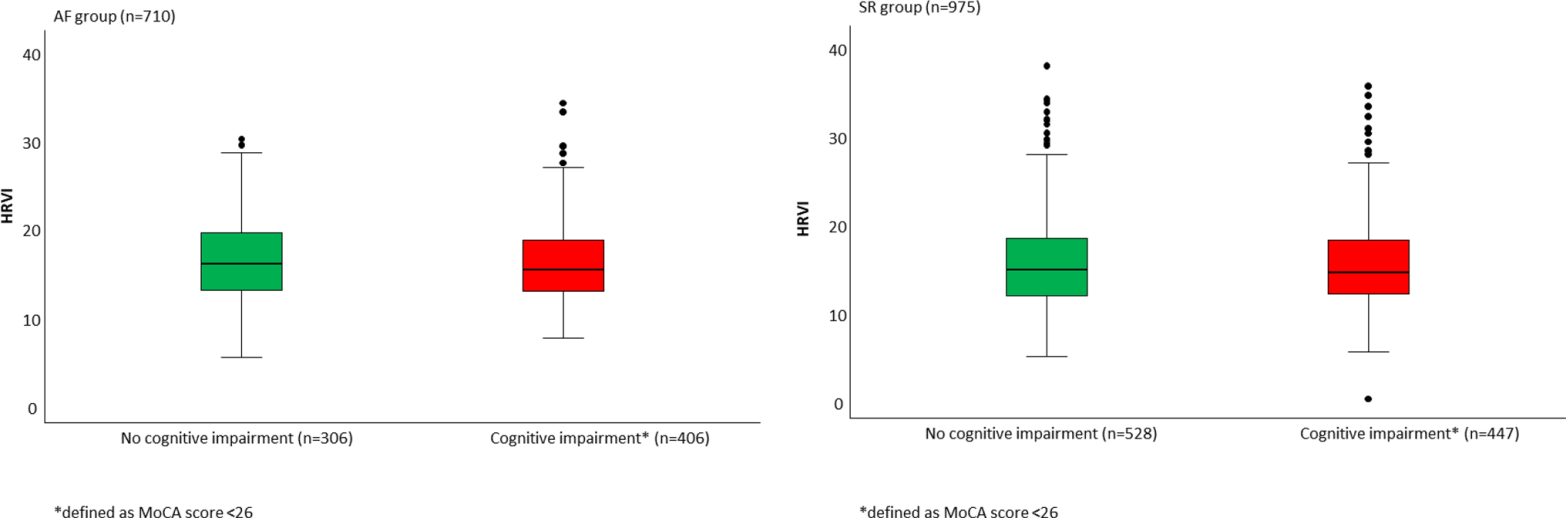
Interdependence of heart rate variability triangular index and cognitive impairment in the sinus rhythm group (left panel) and atrial fibrillation group (right panel)

When patients were categorized into two groups [cognitive impairment (MoCA < 26) vs. no cognitive impairment (MoCA ≥ 26 [15])], median HRVI was lower in patients with cognitive impairment, both in the SR group [14.3 (IQR 11.9–18.0) vs 14.7 (IQR 11.7–18.2), Fig. 3 left panel] and in the AF group [15.1 (IQR 12.7–18.5) 15.8 (IQR 12.8–19.3), Fig. 3 right panel].

We detected no association between MHR and MoCA scores in the SR group (β-coefficient =  − 0.003; 95% CI − 0.007 to 0.002; *p *= 0.254). However, in the AF group, increased MHR was associated with lower MoCA scores (β-coefficient =  − 0.008; 95% CI − 0.014 to − 0.002; *p *= 0.014).

Other HRV parameters (RMSSD, SDNN, 5 min total power, VLF, LF and HF) were not associated with MoCA, neither in the SR nor in the AF group.

## Discussion

In this analysis, we studied the association of CAF with cognitive performance in patients with AF. The main findings of this study are: first, lower HRVI was associated with lower MoCA scores after controlling for covariates, both when measured during SR and during AF. Second, higher MHR was associated with lower MoCA scores in the AF group. Third, RMSSD, SDNN, 5 min total power, VLF, LF and HF were not associated with cognitive function.

Our findings are consistent with results from previous investigations in patients without AF showing that impaired HRV is associated with a decline in global cognitive performance [8–13]. HRV analysis is well established in SR, because the sinus node can be regarded as the “instantaneous writer” of CAF. AF is characterized by highly irregular RR intervals; therefore, most studies analyzing RR interval series have excluded AF patients. However, autonomic factors still may modulate HRV during AF on the level of the AV node [20]. In this analysis, we studied CAF parameters in SR and AF in a population of patients with AF, who are known to be at risk for cognitive decline and dementia [1–5].

Recently, we were able to demonstrate that impaired HRVI, which is a robust measure of overall HRV [18], is an independent predictor of mortality in AF patients [14]. Here, we showed for the first time that lower HRVI is independently associated with a lower cognitive function both when assessed during SR and AF. Beta coefficients seem to be small on an absolute scale, as each 10 units of HRVI lead to a change of 0.5 MoCA points (SR group) and 0.7 MoCA points (AF group), respectively. Nevertheless, this equals approximately a 5-year (SR group) and 7-year (AF group) age difference in cognition, as shown previously [7, 21, 22]. Impaired HRVI is also associated with the presence of silent brain lesions detected by bMRI in the Swiss-AF cohort [45]. Thus, AF patients with low HRVI may be regarded as high-risk patients.

In addition, we found a higher MHR to be independently associated with impaired MoCA scores in the AF group. In AF populations, there is some evidence that a higher heart rate may be associated with progression of AF, incidence of heart failure as well as mortality [23–25]. Similarly, higher heart rates predicted unplanned hospitalizations in a post hoc analysis of pooled data from the AFFIRM and AF-CHF trials [26]. From a pathophysiologic view, the adverse outcomes associated with elevated heart rates may be due to a higher number of isovolumetric contractions, an increase in mean arterial pressure and endothelial dysfunction [23, 26]. Whether lowering the MHR is sufficient to prevent cognitive decline or whether restoring SR by means of rhythm control is currently unknown.

Patients who had AF on the single 5 min baseline ECG recording indicated lower cognitive functioning (approximately1 MoCA point) than patients who presented in SR, independent of age. This may reflect a progress of cognitive decline in more progressive AF types. Previous studies have shown that more progressive disease in AF is associated with more pronounced cognitive dysfunction [27, 28]. Whether this is due to a higher AF burden and possibly a higher rate of resulting structural brain lesions [29], impairment of cerebral perfusion due to reduced cardiac output [30], or due to a higher burden of co-morbidities warrants further investigation.

Numerous factors may contribute to the link between impairment of HRV and cognition. For instance, imbalance of the cardiac autonomic nervous system is associated with poor baroreceptor sensitivity [31, 32]. The baroreceptor reflex ensures a continuous and proper blood flow to various vital organs, including the brain [33]. Impaired sensitivity of the baroreceptor reflex may therefore lead to increased blood pressure variability, which is associated with structural brain damage and a decline in cognition [34–37]. Structural brain lesions, especially right hemispheric or insular stroke, have been associated with HRV depression [38]. Furthermore, neurodegenerative changes may influence cardiac autonomic function via altered autonomic pathways [39]. Finally, cardiovascular risk factors, such as hypertension [40] and diabetes [41], contribute to both cognitive decline and depressed HRV [42, 43].

### Strengths and limitations

The large sample size of a comprehensively characterized and well-treated cohort of patients with AF is a major strength of this analysis. Furthermore, over 1600 MRI scans with detailed information on structural brain lesions were available for this analysis. Therefore, we were able to adjust our multivariable model for the presence of large non-cortical or cortical infarcts. Prior analyses of the Swiss-AF cohort study have shown that the presence of large non-cortical or cortical infarcts are strongly associated with cognition, independently of other structural brain lesions, and irrespective of a known history of stroke or TIA [7]. Some limitations have to be taken into account when interpreting our results. First, CAF cannot be assessed during pacing (about 16% of the Swiss-AF cohort), therefore these patients had to be excluded. Second, results of this analysis are only applicable to short-term ECG recordings (5 min). Therefore, the association between CAF and cognition in AF cohorts should be investigated using 24 h Holter ECG recordings. Third, the results of study should be validated in other AF cohorts. Finally, the cross-sectional and observational nature of the study does not allow to draw conclusions on cognitive decline over time. In other words, no conclusions on causality can be drawn, and confounding effects may still be present.

The association of cardiac autonomic dysfunction and cognitive impairment is well established in cohorts with SR. This is the first study to show that impaired CAF is linked to worse cognitive performance in patients with AF. Particularly, HRVI may be a promising ECG index, as it was independently associated with cognitive dysfunction, irrespective if assessed during AF or during SR and despite being significantly higher in the AF group compared to the SR group. Most recently, the 7th AFNET/EHRA Consensus Conference defined cognitive decline as an important AF-related outcome [44]. AF patients with cognitive impairment may benefit from dedicated neuropsychological interventions and intensive treatment of co-morbidities.

## Conclusion

The present findings provide a new piece of evidence suggesting a positive association of CAF and cognition. However, prospective studies with long-term follow-up are needed to answer the question whether CAF can identify AF patients with risk of future cognitive decline and whether loss in cognition can be prevented by treatment of AF by means of better rate or rhythm control.

## Supplementary Information

Below is the link to the electronic supplementary material.

Supplementary file1 (DOCX 35 KB)
