# Supplementary material for: Cardiac autonomic function and cognitive performance in patients with atrial fibrillation
Source: Clin Res Cardiol. 2021 Jun 22;111(1):60–9. doi: 10.1007/s00392-021-01900-4 (PMC8766386; doi:10.1007/s00392-021-01900-4)
Supplement: Supplementary file 1 — Supplementary file1 (DOCX 35 KB) [file 392_2021_1900_MOESM1_ESM.docx]

**Supplemental Material**

**Cardiac autonomic function and cognitive performance in patients with atrial fibrillation**

**Index**

Swiss-AF investigators 3

Table S1 5

Table S2 6

**Swiss-AF investigators**

University Hospital Basel and Basel University: Stefanie Aeschbacher, Chloé Auberson, Steffen Blum, Leo Bonati, Selinda Ceylan, David Conen, Simone Evers- Doerpfeld, Ceylan Eken, Marc Girod, Elisa Hennings, Elena Herber, Vasco Iten, Philipp Krisai, Michael Kühne, Mirko Lischer, Christine Meyer-Zürn, Pascal Meyre, Andreas U. Monsch, Christian Müller, Stefan Osswald, Anne Springer, Christian Sticherling, Thomas Szucs, Gian Völlmin.

Principal Investigator: Stefan Osswald; Local Principal Investigator: Michael Kühne

University Hospital Bern: Faculty: Drahomir Aujesky, Urs Fischer, Juerg Fuhrer, Laurent Roten, Simon Jung, Heinrich Mattle; Research fellows: Luise Adam, Carole Elodie Aubert, Martin Feller, Axel Loewe, Elisavet Moutzouri, Claudio Schneider; Study nurses: Tanja Flückiger, Cindy Groen, Lukas Ehrsam, Sven Hellrigl, Alexandra Nuoffer, Damiana Rakovic, Nathalie Schwab, Rylana Wenger. Local Principal Investigator: Nicolas Rodondi, Tobias Reichlin

Stadtspital Triemli Zurich: Christopher Beynon, Roger Dillier, Michèle Deubelbeiss,

Franz Eberli, Christine Franzini, Isabel Juchli, Claudia Liedtke, Jacqueline Nadler, Thayze Obst, Jasmin Roth, Fiona Schlomowitsch, Xiaoye Schneider, Katrin Studerus, Noreen Tynan, Dominik Weishaupt. Local Principal Investigator: Andreas Müller

Kantonspital Baden: Simone Fontana, Silke Kuest, Karin Scheuch, Denise Hischier, Nicole Bonetti, Alexandra Grau, Jonas Villinger, Eva Laube, Philipp Baumgartner, Mark Filipovic, Marcel Frick, Giulia Montrasio, Stefanie Leuenberger, Franziska Rutz. Local Principal Investigator: Jürg-Hans Beer

Cardiocentro Lugano: Angelo Auricchio, Adriana Anesini, Cristina Camporini, Giulio Conte, Maria Luce Caputo, Francois Regoli. Local Principal Investigator: Tiziano Moccetti

Kantonsspital St. Gallen: Roman Brenner, David Altmann, Michaela Gemperle. Local Principal Investigator: Peter Ammann

Hôpital Cantonal Fribourg: Mathieu Firmann, Sandrine Foucras, Martine Rime. Local Principal Investigator: Daniel Hayoz

Luzerner Kantonsspital: Benjamin Berte, Virgina Justi, Frauke Kellner-Weldon, Brigitta Mehmann, Sonja Meier, Myriam Roth, Andrea Ruckli-Kaeppeli, Ian Russi, Kai Schmidt, Mabelle Young, Melanie Zbinden. Local Principal Investigator: Richard Kobza

Ente Ospedaliero Cantonale Lugano: Jane Frangi-Kultalahti, Anica Pin, Luisa Vicari Local Principal Investigator: Giorgio Moschovitis

University Hospital Geneva: Georg Ehret, Hervé Gallet, Elise Guillermet, Francois Lazeyras, Karl-Olof Lovblad, Patrick Perret, Philippe Tavel, Cheryl Teres. Local Principal Investigator: Dipen Shah

University Hospital Lausanne: Nathalie Lauriers, Marie Méan, Sandrine Salzmann. Local Principal Investigator: Jürg Schläpfer

Bürgerspital Solothurn: Andrea Grêt, Jan Novak, Sandra Vitelli. Local Principal Investigator: Frank-Peter Stephan

Ente Ospedaliero Cantonale Bellinzona: Jane Frangi-Kultalahti, Augusto Gallino. Local Principal Investigator: Marcello Di Valentino

University of Zurich/University Hospital Zurich: Fabienne Witassek, Matthias Schwenkglenks.

Medical Image Analysis Center AG Basel: Jens Würfel (Head), Anna Altermatt, Michael Amann, Petra Huber, Esther Ruberte, Tim Sinnecker, Vanessa Zuber.

Clinical Trial Unit Basel: Michael Coslovsky (Head), Pascal Benkert, Gilles Dutilh, Milica Markovic, Pia Neuschwander, Patrick Simon

Schiller AG Baar: Ramun Schmid

Table S1: Associations between time and frequency domain measures of HRV and the study endpoint (MoCA) when additionally adjusted for AF duration, non-dihydropyridine calcium channel blockers and digoxin

| HRV parameter | Sinus rhythm group | | Atrial fibrillation group | |
| --- | --- | --- | --- | --- |
|  | β (95% CI) | p-value | β (95% CI) | p-value |
| HRVI | 0.050 (0.017; 0.082) | 0.003 | 0.068 (0.020; 0.116) | 0.006 |
| MHR | -0.003 (-0.007; 0.002) | 0.236 | -0.008 (-0.014; -0.002) | 0.016 |
| RMSSD | -0.008 (-0.018; 0.001) | 0.079 | -0.007 (-0.016, 0.003) | 0.164 |
| SDNN | 0.001 (-0.002; 0.003) | 0.564 | -0.001 (-0.006; 0.004) | 0.750 |
| 5-minute total power* | 0.094 (-0.139; 0.328) | 0.428 | -0.072 (-0.813; 0.669) | 0.849 |
| HF* | 0.044 (-0.198; 0.286) | 0.720 | 0.014 (-0.630; 0.659) | 0.965 |
| LF* | 0.003 (-0.217; 0.224) | 0.972 | -0.155 (-0.798; 0.493) | 0.637 |
| VLF* | 0.044 (-0.176; 0.263) | 0.685 | -0.293 (-0.653; 0.060) | 0.103 |

*log-transformed. Multivariable model was adjusted for age, sex, body mass index, smoking status (current/past vs. never), alcohol consumption, presence of large noncortical or cortical infarcts, history of hypertension, history of diabetes, education (basic, middle, advanced), history of oral anticoagulation therapy, AF duration, intake of betablockers and/or class Ic/III antiarrhythmics, calcium-channel blockers and digoxin. HF = high frequency. HRV = heart rate variability. HRVI = heart rate variability triangular index. LF = low frequency. MHR = mean heart rate. MoCA = Montreal Cognitive Assessment. RMSSD = the square root of the mean squared differences of successive normal-to-normal intervals. SDNN = standard deviation of the normal-to-normal intervals. VLF = very low frequency.

Table S2: Associations between time and frequency domain measures of HRV and the study endpoint (MoCA) by the use of Tobit regression

| HRV parameter | Sinus rhythm group | | Atrial fibrillation group | |
| --- | --- | --- | --- | --- |
|  | β (95% CI) | p-value | β (95% CI) | p-value |
| HRVI | 0.049 (0.016; 0.080) | 0.004 | 0.070 (0.022; 0.118) | 0.005 |
| MHR | -0.002 (-0.008; 0.004) | 0.297 | -0.007 (-0.010; -0.004) | 0.012 |
| RMSSD | -0.009 (-0.019; 0.001) | 0.071 | -0.007 (-0.017; 0.003) | 0.169 |
| SDNN | 0.001(-0.002; 0.004) | 0.553 | -0.001 (-0.007; 0.004) | 0.727 |
| 5-minute total power* | 0.104 (-0.142; 0.342) | 0.397 | -0.085 (-0.866; 0.690) | 0.822 |
| HF* | 0.046 (-0.210; 0.294) | 0.715 | 0.008 (-0.672; 0.696) | 0.981 |
| LF* | 0.013 (-0.229; 0.241) | 0.907 | -0.165 (-0.824; 0.473) | 0.616 |
| VLF* | 0.044 (-0.201; 0.260) | 0.705 | -0.277 (-0.718; 0.098) | 0.129 |

*log-transformed. Multivariable model was adjusted for age, sex, body mass index, smoking status (current/past vs. never), alcohol consumption, presence of large noncortical or cortical infarcts, history of hypertension, history of diabetes, education (basic, middle, advanced), history of oral anticoagulation therapy, intake of betablockers and/or class Ic/III antiarrhythmics. HF = high frequency. HRV = heart rate variability. HRVI = heart rate variability triangular index. LF = low frequency. MHR = mean heart rate. MoCA = Montreal Cognitive Assessment. RMSSD = the square root of the mean squared differences of successive normal-to-normal intervals. SDNN = standard deviation of the normal-to-normal intervals. VLF = very low frequency.
